# The role of traditional healers in tooth extractions in Lekie Division, Cameroon

**DOI:** 10.1186/1746-4269-7-15

**Published:** 2011-05-30

**Authors:** Ashu M Agbor, Sudeshni Naidoo, Awono M Mbia

**Affiliations:** 1 Department of Community Dentistry, University of the Western Cape, Tygerberg, South Africa; 2 Department of Dentistry. Regional Hospital, Bamenda, Cameroon

## Abstract

**Background:**

The extraction of the teeth by traditional healers in Cameroon is an established cultural practice in the central region of the Cameroon. Traditional healers (TH) use herbs and crude un-sterilized instruments and tools for the tooth extraction procedure. The present study investigates the knowledge and practices of traditional healers regarding tooth extraction and the management of its complications.

**Methods:**

A cross sectional design utilizing semi-structured questionnaires was used to collect the data from traditional healers and their patients.

**Results:**

Sixteen traditional healers (TH) were interviewed. All were male and the majority were between 25-35 years old. The most important reason given for the removal of a tooth was "if it has a hole". All reported using herbs to control bleeding and pain after extractions. Only 20% used gloves between patients when extracting a tooth and just over a third (31.3%) gave post-operative instructions. Eighty seven percent managed complications with herbs and 62.5% reported that they would refer their patients to a dentist whenever there are complications. Only a third (31.3%) was familiar with the basic anatomy of a tooth and more than half (56.3%) reported that tooth extractions are the only treatment for dental problems.

One hundred and fifty patients were interviewed with a mean age of 29 years. More than two thirds were in the 21-30 year age group and just over half were male. Sixty six percent reported that they visited the TH because it is cheap, 93.3% were satisfied with the treatment they received while 95.3% reported said they never had a problem after an extraction.

**Conclusions:**

Tooth extractions using medicinal plants is well established in Lekie division, Cameroon. Infection control during extraction is not the norm. Traditional healers are willing to co-operate with oral health workers in improving the oral health of their patients. Mutual cooperation, collaboration and integrating TH into primary oral health care services need to be increased.

## Background

Tooth extraction is an ancient practice that is carried out worldwide. It has been a common practice of traditional healers (TH) in sub-Saharan Africa for centuries [1]. The most common reasons for tooth extraction documented in Africa are ritual tooth extraction and infant tooth mutilations [1]. Apart from mutilations, extractions are carried out for superstitious, aesthetic or other reasons depending on culture and tradition. In the West, evidence of these practices was first noticed among slaves transported from sub-Saharan Africa to the new world in the early 18^th ^century [2]. Southern Africa has a long history of dental mutilation in the form of dental chipping and of intentional removal of anterior teeth [3]. Early evidence from many archaeological sites in the Southern Africa was found in the skeletons of Early Iron Age populations (ca. 1500 years before present)[3].

Traditional healers in Africa have been carrying out surgery ranging from circumcision during initiation ceremonies, traditional autopsy and tooth extractions based on socio-cultural beliefs [4]. In South Sudan, itinerant TH have been reported to perform surgery for tooth extraction, abortion, incision and drainage of abscesses, uvulectomy, circumcision, inguinal hernia surgery, non-invasive cataract luxation and closed and open fractures [4]. In Somalia, it has been reported that tooth extractions constitute thirty eight percent of the surgical procedures carried out by traditional healers [5]. Although ritual dental extraction among Sub-Saharan African populations has been practiced for centuries, little is known about the practice in other ethnic groups particularly among adult populations [1].

Like most sub-Saharan countries, traditional healers in Cameroon are ubiquitous [6,7]. They are often the first point of contact for those seeking health care [6,7]. Most people rely on TH because their treatment is affordable, they share patient's culture, beliefs and values and understand their expectations of health care [7,8]. People prefer TH because they are more accessible and acceptable than other health care providers in their communities [8]. In addition, most people have the perception that TH methods of treatment are more effective and less invasive since they utilize herbs and medicinal plants [7].

Traditional healers thus play an important role in the delivery of primary health care, particularly in remote communities [6-9]. Puckree and colleagues [7] in KwaZulu Natal, South Africa reported that about 70% of patients consulted a traditional healer as a first choice for health care including potentially life-threatening conditions [7]. They concluded that since oral health care was an integral component of health care in South Africa, health care professionals needed to be proactive in integrating traditional healing with westernized practices in order to promote health for all [7].

The aim of the present study was to assess the knowledge and practices related to tooth extraction by traditional healers TH in Lekie division of Cameroon. The objectives were to investigate the practice of tooth extractions, to determine why clients patronise TH and how complications associated with tooth extraction are managed.

## Methodology

The study was carried out in Ebo-Ndeg, a village in the Lekie Division. Lekie division (a typical administrative unit in Cameroon) is a highly populated rural area with a population of 500500 people and a population density of 169 people per square kilometre. Monatele its headquarters, is 49 km from Yaounde, the capital of Cameroon. There is no dentist or oral health care personnel in this area and there are 312 TH registered with the TH association. They treat common problems like diarrohea and malaria to more complex cases like bone fractures. The number of TH involved in tooth extractions is unknown.

The population is dominated by the Eton (70%) and Ewondo (30%) ethnic groups. Eton and Ewondo are 2 sub-ethnic groups from the Beti tribe, a Bantu tribe that inhabit the equatorial forests covering the forest regions of Equatorial Guinea, Gabon and Congo Brazzaville. Most people in this area rely on subsistent agriculture but a large part of the land has cocoa plantations which produces cocoa as the main cash crop.

Data was collected from sixteen traditional healers and one hundred and fifty (150) patients. The study consisted of two parts: TH who carry out tooth extraction were observed and interviewed thereafter. A convenience sample of patients (adults 20 years and above) were recruited as they visited the TH for treatment or follow-up. The TH were chosen by the village heads. Informed consent was obtained from all TH and patients.

### Procedure

**Figure 1:**
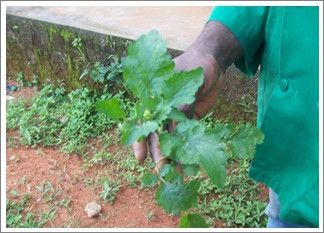
**TH harvesting medicinal Plant (*Dichrocephala intergrifolia)***.

**Figure 2:**
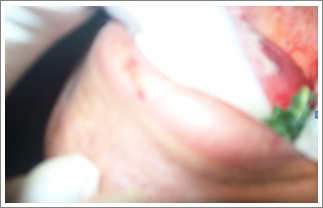
**Application of plant to infected tooth**.

**Figure 3:**
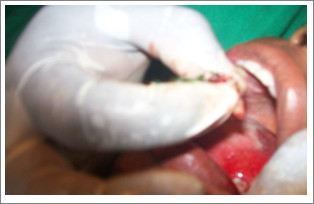
**Extraction of tooth by TH**.

Following consent, the researcher and an assistant accompanied the TH to harvest the plant that was to be used for the extraction [Figure 1]. The TH squeezed the juice from the plant and applied the whole of it on the tooth for 3 minutes [Figure 2]. Thereafter, with his bare hands and nails pulled the tooth out of the socket [Figure 3]. After removal of the tooth, the researcher observed how the TH controlled the bleeding and whether any post-operative instructions were given. Patients were observed during the extractions for signs of pain or discomfort from their facial expression and eyes. After the procedure, a questionnaire was administered to the TH. Information was obtained on the knowledge of tooth extraction, the materials used, knowledge of the anatomy of the tooth, post extraction instructions, management of complications and prevention of infection. Questionnaires were administered to patients immediately after treatment.

A sample of the plant was collected and was taken to the Department of Botany, University of Dschang (Cameroon) for identification and classification.

Ethical clearance was obtained from the Ministry of Higher Education and Scientific Research. Verbal authorisation was obtained at each study site from the head or chief of the village.

Data were categorized, coded and then entered into the computer. The data was captured in Excel. Basic descriptive analysis was done using the Excel environment. The database was imported into Epi-info ^® ^version 3.5.2 to perform complex statistical analyses. Descriptive statistics were used to describe the demographic factors.

## Results

### Traditional Healers

Sixteen TH were interviewed. All were male and the majority were between 25-35 years old. The age range was 26-65years. Ten (62.5%) were in practice full time, 4 (25.5%) part-time TH and farmers and 2 (12.3%) were civil servants.

The majority reported that they will extract the tooth if it "has a hole". Other reasons included fracture of the tooth and when the patient requested an extraction [Table 1]. Nine (60%) used their hands, fingers and fingernails for extractions, 3(20%) scissors, 2(13.3%) broken glass and 1(6.7%) a sewing needle. More than half ((56.3%) reported that they knew that the extraction was successful when the both the crown and roots are removed [Table 2].

**Table 1:** Assessment of a tooth to be extracted

How do you know that the tooth needs to be extracted?	Percent
Has a hole	37.5%
Patient has pain	18.8%
Tooth is shaking, loose in the socket	12.5%
Tooth roots are retained	6.3%
Other	25.0%
**Total**	100.0%

**Table 2:** Assessment of complete tooth extraction

How do you know that the tooth is completely removed?	Percent
When crown and roots are removed	56.3%
When the crown is seen	31.3%
When one root is seen	12.5%
**Total**	100.0%

Only 20% change gloves between patients when carrying out a tooth extraction and just over a third (31.3%) gave post-operative instructions. All reported using herbs to control bleeding and pain after extractions. The majority (87.5%) managed complications with herbs and two thirds (62.5%) reported that they would refer their patients to a dentist whenever there are complications. Only a third (31.3%) reported that they were familiar with the basic anatomy of a tooth (referring to the crown the 'head' of the tooth and the roots the 'legs') and 75% could correctly define "tooth extraction". More than half (56.3%) reported that tooth extraction is the only treatment for dental problems.

### Patients

One hundred and fifty patients were interviewed. More than two thirds in the 21-30 year age group and a mean age of 29 years. Just over half were male (51.3%). More than three quarters (84.7%) were from the rural areas.

Tooth extractions (81.3%) was reported to be the most common form of treatment provided followed by pain reduction (17.3%).

More than two thirds (66.7%) reported that they visited the TH because it is cheap [Table 3]. The majority (93.3%) were satisfied with the treatment they received and 95.3% reported said they have never had a problem after an extraction. Reported problems included infection in the extraction socket and jaw swelling.

**Table 3:** Reasons for visiting a TH for dental problems

Why do you visit a traditional healer for tooth problems?	Percent
Cheap	66.7%
Fast treatment	16.0%
Efficient (painless) treatment	12.7%
Easier access	4.7%
**Total**	100.0%

## Discussion

The present study describes tooth extractions using a medicinal plant (*Dichrocephala integrifolia) *in the Central Region of Cameroon. It also assessed the knowledge and practice of TH involved in this practice. To the best knowledge of the researchers, this is the first study reporting the use of a medicinal plant by TH for tooth extractions in Cameroon.

Extractions are carried out using the fresh leaves and stems of *Dichrocephala integrifolia*, one of the natural plants used for atraumatic tooth extraction in Cameroon. This plant is a common shrub that grows domestically and in the wild. It is called "Mbag'api" in Bameleke a tribe in the western region and also called "Ngnignada Elok" in Ewondo language, one of the local dialects spoken in Lekie division of Cameroon which means, "Remover". The plant is placed on the fractured, painful or carious tooth and left for about two-three minutes. The tooth becomes loose and is pulled out with fingers or any sharp instrument. Thereafter the plant is put into the extraction site for about an hour to enhance clotting and arrest bleeding. No trauma or pain is involved. Because of the anaesthetic properties of the plant, no local anaesthesia is used.

### Traditional healers

The extraction of the teeth by traditional healers in Cameroon is an established cultural practice in the central region of the Cameroon. In the Lekie Division, Cameroon it has been a tradition that has been passed down from one generation to another. This culture of traditional tooth extraction has been confirmed by dental arch alterations due to tribal mutilations in a 14^th ^century skull from Cameroon [10].

Traditional healers use herbs and crude un-sterilized instruments for the tooth extraction procedure. They do not follow universal infection control measures regarding tooth extractions. Management of dental problems with herbs have been reported in other areas of Africa. Ngilisho and colleagues reported on the role of TH in the treatment of toothache in Tanga region, Tanzania and found that sixty per cent of villagers that suffered from toothache sought treatment from TH [9]. They were treated with local herbs and forty per cent who sought this service obtained relief of pain for more than six months. Several cases have been reported in Africa on practices that involve the removal of the tooth germs or infant oral mutilation (IOM) [9,11-13]. The practice of extracting tooth buds and of rubbing herbs on to the gingivae of children to treat fever and diarrhoea, has been documented in countries like Tanzania and Uganda [11,12] despite the fact that these are not scientifically proven treatments [13].

In the present study the diagnosis of dental pathology was mostly by visual examination. Other studies in Africa have reported that most traditional healers diagnose toothache in this way without palpation of the soft and hard tissues. Others use the clients' history of pain or obtain the cause of the pathology from a previous case of extraction [14,15]. Traditional healers therefore have little or no training in oral diagnosis and anatomy as compared to conventional dental practitioners who have been trained in head and neck anatomy, tooth anatomy as well as the progression of dental disease. Trained practitioners use the extent and depth of the carious cavity, the surface area it covers and the nature of the pain as important diagnostic criteria for the management of a decayed tooth. Nowadays, minimal intervention dentistry advocates conservation of as much of the sound tooth structure as is possible and emphasises that extraction of the tooth should be the last resort after root canal and other restorative treatments.

The present study found that most of traditional healers who carry out tooth extraction have little knowledge of tooth anatomy or pathology related to tooth decay and this often resulted in extraction of teeth that may have been preserved through restorative or endodontic treatment. Only 31.3% were familiar with the basic anatomy of the tooth - referring to the crown as the "head" and the roots as the "feet" of the tooth. Rampant tooth extraction could be one of the reasons for the high prevalence of partial edentulism in this region. A study carried in Sangmelima, a town close to Yaoundé, found that 50.8% of the population needed artificial teeth [16]. High edentulism can also be reduced if TH are taught to carry out basic restorative techniques such as the Atraumatic Restorative Treatment (ART). This has been proposed in a previous study carried out in Bui Division of Cameroon [8]. Traditional healers need to collaborate with oral health workers to improve their knowledge on the basic anatomy of the tooth so as to properly diagnose and refer cases that the tooth can be preserved or saved.

More than half of the TH affirmed that an extraction was deemed successful when there was complete removal of the crown and roots. However, in the course of the extraction process, retained roots may go unnoticed as some TH lack basic knowledge of tooth anatomy. Besides, the strong analgesic effect of the plant could mask post-extraction pain which usually results from retained roots. Much needs to be improved in this area and TH involved with tooth extractions will benefit greatly from basic training.

In this study, two thirds of TH used their fingers and fingernails to extract the loosened tooth from its socket, while others used unsterile objects like broken glass, scissors and sewing needles. Other studies have reported that TH used unsterilized tools to excise tooth follicles without local anesthetic. In addition to knives and fingernails, the healers used "tools" like bicycle spokes, rusty nails or wire [17,18]. Due to the fact that no standard infection control methods are used, it can surmised that tooth extractions pose a major risk for the transmission of infectious diseases. Other invasive traditional surgical practices like male circumcision, tattooing, scarifications and female genital mutilations have also been identified as major risk factors for HIV transmission when tools are re-used without sterilization [18-20]. Further studies have shown that many TH expose themselves and their patients to infections in the course of their practice. Traditional healers from Zambia and Botswana use unsterile sharp instrument in treating patients and some use their mouth to suck blood (blood letting) from their patient's body as part of disease management, and in some cases use their bare fingers to transfer mufti (traditional medicines) from one patient to another [21,22].

Post-operative complications in this study were low despite the fact that only a third of TH provided post-operative instructions. Routinely, post-operative instructions are given to prevent trauma and complications that may arise like pain, bleeding, infection and necrosis of extraction socket (dry socket). Most TH managed complications with herbs. All TH used the same herbs for tooth extractions and for the control of post-operative bleeding and pain.

### Use of plants

*Dichrocephala intergrifolia *has been shown to possess anti-inflammatory and analgesic properties and has been used to treat cattle for swelling, infection, necrosis, oedema and pain [23]. However, the anti-inflammatory and anti-haemorrhagic properties on humans need to be investigated further. The use of plant materials for tooth extraction have been reported for example in the Trio tribe in Latin America, where the soft wood of the *lapa lapa *tree is applied to the gums, causes swelling of the gingivae and assists to dislodge the root prior to extraction [24]. The use of herbs as an adjunct to dental treatments like tooth extractions and the management of some dental problems has been documented. Herbs have been reported to have anti-inflammatory, analgesic, antimicrobial and anti-hemorrhagic (haemostatic) properties [25-29].

*Solanum torvum *(family Solanaceae) also known as 'top na aka' in the Batoufam language, is a plant used in Cameroonian folk medicine for the treatment of fever, wound healing and tooth decay [25]. It is reported to have anti-microbial, anti-viral [26,27] and haemostatic properties [27]. A study carried out in Peru on 510 plants showed that 11 species were identified for the treatment of infection, 59 species had anti-inflammatory properties and 43 were used to treat wounds and had haemeostatic properties [28]. In southern Peru and Northern Ecuador 5 species (1%) of plants identified in another study were found to be used in the treatment of general pain, intense body pain (e.g., caused by dengue fever), as well as tooth pain and post-operative pain after tooth extraction. Thirteen species were reportedly used as analgesics for the treatment of headaches, general pain and toothache in Ecuador [29].

The immature polycarp of *Gamipa Americana *(family Rubiaceae) is used for tooth extraction by placing the pulp of the plant onto the aching tooth, where it was left in place for several weeks causing disintegration of the tooth which is then removed in pieces, with little or no trauma [30]. The stem-sap of *Stigmaphyllon *species (family Malpighiaceae) is placed on the carious tooth for about four hours followed by repeated applications throughout the day. After one week, the tooth can be removed without bleeding or pain [31]. In Guatemala, boiled tree bark, herbs and camphor are used for the treatment of tooth and head pain [32]. *Daceryodes excelsa *is thought to be the herb widely used to loosen teeth prior to extraction and its resin alleviates toothache and loosens the roots of a dead tooth [33,34].

The aggressive stinging ants found inside the stems of *Triplans *species (family Polygonaceae) are crushed and placed on the aching tooth for one week. The tooth is then pulled out with the fingers. It is thought that formic acid (among other substances) in the stinging ants is responsible for the loosening of the tooth [14]. One repeated application of swabs of the latex of *Chlorophora tinctora *(family Moraceae) is also used for tooth extraction. No pain, trauma or bleeding is involved [15]. Careless application often results in spillage or damage to other teeth and may lead to unintended extraction of unaffected teeth [35].

### Complications of traditional medical practices

Eduard's et al. [36] reported on adverse effects (immediate, short-term complications and long-term psychological and dental side effects) of traditional medical practices in children. In the period immediately after the procedure, the most common risks include excessive bleeding, infection, osteomyelitis of the jaws, noma, tetanus, meningitis, aspiration bronchopneumonia, transmission of infectious diseases (including HIV and hepatitis) and death. No statistics are available regarding adult morbidity and mortality related to complications of traditional tooth extraction practices [36].

A high prevalence of complications has been reported among children with the ebino and tea-tea procedures in Tanzania [11]. They include haemorrhage, septicaemia, tetanus, gangrene, contractures, abscesses, airway obstruction, iatrogenic fistulae, laceration of vital organs and death [9]. Harmful practices include the use of penknives, metal blades made from spoon handles or bike tire spokes and fingernails [18,19]. In some cultures, salt or herbs are applied to the area of the gum that is injured following the procedure [11,37]. In Uganda post-ebino extraction complications included septicaemia, anaemia, difficulties in feeding and pain. Some children required hospitalization [13]. The practice has other consequences - infections at the site of the procedure and death from sepsis have been documented in a number of studies [38,39].

HIV infection is reported to be one of the complications. Physicians working in Africa report that traditional practitioners will carry out surgical procedures on several successive children in a short period of time using the same instrument [40]. This practice is common in areas of Kenya, where nearly a third of the pregnant women are HIV-positive, suggesting a possible mechanism for the horizontal spread of the virus [41]. In Tanzania, TH have been reported to use cutting and injection equipment on up to 10 patients in a single clinic session. These procedures cause haemorrhage, septicaemia, tetanus, gangrene, contractures, abscesses, airway obstruction, keloids, iatrogenic fistulae, lacerations of vital organs, loss of limbs, and death [9].

An Ethiopian study showed decreased growth in children who had dental extractions as long as 4 months after the extractions, and this reduction was observed even when controlling for illness episodes [39]. Perhaps the most important consequence of this practice is that the children are not correctly diagnosed or treated for their febrile or gastrointestinal illnesses because parents either do not return to health care facilities or seek care directly from a traditional healer [40,41].

Apart from poor nutrition as a result of tooth loss [42], other long term effects of partial edentulism include individual psychological effects especially when individuals expressed embarrassment about their dental status, which limit smiling, speaking and social interaction [42]. Malocclusion has been reported as one of the complications of early unguided extractions. A survey carried out in Kampala to determine the occlusal traits of 402 fourteen-year-old children on the effects of ebinyo (a dental mutilation) on the occlusal status children showed that the practice of ebinyo (although carried out early in the life of the child) can impact on the occlusal status in the permanent dentition years later [43].

### Patients

In the present study, the majority of patients were male. This is in contrast to similar studies carried out in urban centres in the same province [20,44,45]. In general, caries prevalence is higher among women and more women in urban areas have been reported to visit dental clinics [20]. This is thought to be due to their higher levels of education. Furthermore, younger women visit hospitals and clinics for other health needs and with their children, so may they use this opportunity to visit the dental clinic. In rural areas, women are poor and uneducated and depend on their husbands for income and may not have easy access to any form of health care.

A study carried out in Cameroon [8] reported that two thirds of patients, who visit TH at Bui division of Cameroon, were aged between 21-40 years and this was attributed to the demographic profile of the area since a larger proportion of the population is young and lives in rural areas. In the present study, the majority of patients were aged between 21 and 30 years and their nutritional habits may have predispose them to increased dental disease due to the changing and more cariogenic diet in Africa [46]^.^

Tooth extraction was the most common form of dental treatment provided. Other treatments reported included the management of gingivitis, oral ulcers and some oral HIV lesions. In Cameroon, TH have been reported to treat oral candidiasis, dental caries and gum diseases using mouth washes made from the barks of trees, herbs and roots [8,47].

Apart from the fact that treatments offered by TH are cheap and that they are easier to access, most patients confirmed that they visited TH because their treatments were painless and faster. This has a psychological impact on the patients [41] as anticipated pain during dental treatment causes anxiety. It has been found that patients with high dental anxiety are likely to have exaggerated memory and prediction of dental pain [48]. Furthermore, the cost of restorations and other conservative care like root canal treatment is also very high and is an additional reason why more patients patronise TH. Traditional medicine is the first choice health care treatment for at least 80% of Africans who suffer from fever and other common ailments [31].

## Concluding Remarks

The extraction of the teeth by traditional healers in Cameroon is an established cultural practice in the central region of the Cameroon. This study has shown that traditional healers use herbs and common house items to carry out tooth extractions. They use local or medicinal herbs like *Dichrocephala intergrifolia *instead of local anaesthetic solution to numb the area. They do not follow universal infection control measures regarding the routine use gloves, aprons, face masks, sterilizers. However, they do and can act as primary health care providers in areas where there is no oral health care facility.

## Recommendations

The World Health Organization has developed models for institutionalizing African medicine in health systems. The models are available to those who need them and may be adopted or adapted to suit local situations [22].

**1.**	Promote and conduct collaborative programmes of training of traditional healers who carry out dental extractions. Instruction should be provided on tooth anatomy, diagnoses, standard infection control measures and the atraumatic restorative techniques (ART).

**2.**	 Institute intensive education on the management of post-operative complications.

**3.**	Promote and conduct scientific research on the traditional medicinal plants used for tooth extraction.

**4.**	The Ministry of Public Health should enhance and support the co-operation and collaboration between traditional healers and dental practitioners so as to encourage referral.

**5.**	The Ministry of Higher Education and Scientific Research should identify TH and utilise them to assist with the standardisation of their practice.

**6.**	The mechanism of action, adverse effects and other medical applications of *Dichrocephala intergrifolia *should be studied.

## Competing interests

The authors declare that they have no competing interests.

## Authors' contributions

AMA contributed to the design and conception of the study as well as acquisition of data, its analysis and interpretation and was involved in the drafting of the manuscript. SN made substantial contributions to the design and in the drafting, revision and finalization of the manuscript.AMM contributed in the design and acquisition of data. All authors read and approved the final draft of the manuscript.
